# The Association between Adult Sport, Fitness, and Recreational Physical Activity and Number and Age of Children Present in the Household: A Secondary Analysis Using NHANES

**DOI:** 10.3390/ijerph20115942

**Published:** 2023-05-24

**Authors:** Jerraco L. Johnson, Ailton Coleman, Jamila L. Kwarteng, Ahondju U. Holmes, Dulcie Kermah, Marino A. Bruce, Bettina M. Beech

**Affiliations:** 1 Department of Kinesiology, Health Promotion, and Recreation, University of North Texas, Denton, TX 76203, USA; 2 Department of Health Sciences, James Madison University, Harrisonburg, VA 22807, USA; colemaas@jmu.edu; 3 Division of Community Health, Institute for Health and Equity, Medical College of Wisconsin, Milwaukee, WI 53226, USA; jkwarteng@mcw.edu; 4 University of Oklahoma Stephenson Cancer Center, Oklahoma City, OK 73014, USA; ahondju-holmes@ouhsc.edu; 5 Urban Health Institute Student Research Core Charles R., Charles R. Drew University of Medicine and Science, Los Angeles, CA 90059, USA; dulciekermah@cdrewu.edu; 6 Department of Health Systems and Population Health Sciences, University of Houston Tilman J. Fertitta Family College of Medicine, Houston, TX 77021, USA; mabruce@central.uh.edu (M.A.B.); bmbeech@central.uh.edu (B.M.B.); 7 UH Population Health, University of Houston, Houston, TX 77021, USA

**Keywords:** children health, parent health, family health, moderate-to-vigorous physical activity (MVPA), cardiovascular health

## Abstract

Only one in three adults in the United States meets the weekly recommendation for physical activity (PA). The presence of children in the home may restrict adult PA. The purpose of this study was to examine the association between adult moderate and vigorous sport, fitness, and recreational physical activities and the number and age (0–5 and 6–17) of children in their household. Secondary data were drawn from the National Health and Nutrition Examination Survey (NHANES) from 2007–2016. Adults with complete survey data for self-reported moderate (MPA) and vigorous physical activities (VPA), number of children in the home, and other sociodemographic variables were included. The final sample included 2034 adults from 22–65 years of age. Analyses included ANOVAs and separate multivariable regression analyses to determine if the number of children in the household aged 0–5 and 6–17 were significant predictors of weekly moderate-to-vigorous physical activity (MVPA) after controlling for covariates. For MPA, no differences were found between adult PA regardless of the number and age of children in the home. For VPA, adults with two or more children aged 0–5 reported 80 fewer minutes of weekly VPA (*p* < 0.05) compared to those with no children or just one child in this age group after controlling for all covariates. Finally, adults with three or more children in the household aged 6–17 reported fifty fewer minutes of weekly VPA (*p* < 0.05) compared to those with no children, one, or just two in the household. These findings highlight a need to support the vigorous PA behaviors of this population, as the majority of the family-based PA intervention studies to date have primarily focused on family dyads.

## 1. Introduction

Engagement in regular physical activity (PA) is a critical health behavior across the entire lifespan. The recent *Physical Activity Guidelines (PAG) for Americans,* 2nd edition [1] identified new benefits of physical activity for individuals of all ages, including healthy weight maintenance for young children, improved cardiometabolic health during adolescence, and a lower risk of cardiovascular disease and all-cause mortality among adults [2]. Despite these benefits, an overwhelming majority of adults in the United States (U.S.) do not engage in regular physical activity. In 2020, only 47.0% of adults in the U.S. adhered to the recommended aerobic PA guidelines of at least 150 min of moderate PA and 75 min of vigorous PA per week [3]. Overall, this lack of physical activity is associated with nearly USD 117 billion in annual health care costs, which highlights a significant financial implications for the U.S. [1].

Parenthood is associated with a decrease in PA and a concurrent increase in weight gain [4], which increases the risk for chronic diseases. Several studies report that parents are less physically active compared with adults without children [5,6,7]. More importantly, reduced PA may be transmitted to children, as parents are likely to serve as role models for their children’s PA behaviors [8,9,10,11]. Similar to adult physical activity behaviors, only 24% children 6 to 17 years of age engage in the recommended 60 min of daily physical activity [12], with a significant decrease in PA between children aged 6–11, 12–15, and 15 and older. Katzmarzyk et al. [13] created a physical activity report card for U.S. children and youth and assigned an extremely poor grade of a D- based on the low levels of overall physical activity and high levels of sedentary behaviors of children and adolescents. Thus, understanding the factors associated with the PA behaviors of parents may be critical for both parents and children.

### Parenthood and PA

The number of children in the household, age of children, and how children may impact mothers and fathers differently have all been associated with time spent in sedentary, light, and moderate-to-vigorous physical activity (MVPA) by parents. In a nationally representative U.S. sample, Carson et al. [5] found mothers to be less active than women without children regardless of the number of children and age of the youngest child. Interestingly, this relationship did not hold true for fathers in the same study. Gaston et al. [6] reported similar findings in a Canadian sample and found a stronger relationship between PA and parenthood for women versus men. However, unlike the U.S. sample, both the number of and age of the children in the household significantly impacted the mothers’ PA, as women with multiple children and younger children under the age of six engaged in significantly less MVPA compared to non-parents and those with older children. These findings are aligned with those of Adamo et al., [14] who also reported that the age of the child may impact the PA of mothers and fathers differently. They found that mothers with children under the age of six engaged in one less hour of weekly MVPA compared to those without children, yet the fathers’ MPVA was more impacted by older children aged six to eleven compared to younger children [14]. Conversely, a longitudinal study by Hull et al. [7] found fathers’ PA to be more impacted than mothers’ during the initial transition to parenthood (e.g., during the first two years of life). In fact, they reported no differences in PA changes between women who were childless and those that transitioned into parenthood. Moreover, having a subsequent child during this 2-year period (i.e., having two infants under two) was more detrimental to the mothers’ PA compared to fathers’.

From a societal perspective, it appears that mothers’ PA may initially be impacted based on normative gender roles, as women tend to spend more time on childrearing than men for young infants [15]. In this regard, few studies have examined the impact the age of children has on parental PA in more depth [4,16]. Berge et al. found that young adult mothers and fathers with children under the age of one engaged in less MVPA than adults without children [4]. More recently, in a UK sample, Simpson [16] found that women with younger children under age 4 engaged in less MVPA compared to mothers with school-age (older) children. They also found that mothers with more than one child engaged in less MVPA than those with just one child. They concluded that mothers with multiple children and young children engage in less MVPA than non-mothers and those with only one child. While these studies seem to agree that time spent in PA is different for parents compared to non-parents, a few studies have reported contrasting findings. Nomaguchi and Bianchi [17] examined the relationship between the number of children in the household and their exercise time. Based on self-reported measures of PA, they found no differences in exercise time based on the number of children an individual had. Candeleria et al. [18] reported similar findings in a Canadian sample. They found no significant differences in objectively measured MVPA based on parent status or number of children in the household amongst adults. While there were no differences in MVPA, they did find differences in sitting time and light household PA between parents and non-parents.

Collectively, these findings indicate that parenthood can reduce physical activity engagement, but mixed findings have been reported regarding the association between the number and age of children and moderate and vigorous physical activity (MVPA). This study will begin to address this gap by examining the relation between adult moderate and vigorous PA based on the number and age of children in the home using a nationally representative sample in the U.S. The purpose of this study was to examine the association between adult moderate and vigorous sport, fitness, and recreational physical activities, and the number and age (0–5 and 6–17) of children in their household. We hypothesized that adult PA would decrease relative to the number of children in the household, and that this relationship would be stronger for young children aged 0–5 versus adolescents aged 6–17.

## 2. Materials and Methods

### 2.1. Study Sample

**Figure 1:**
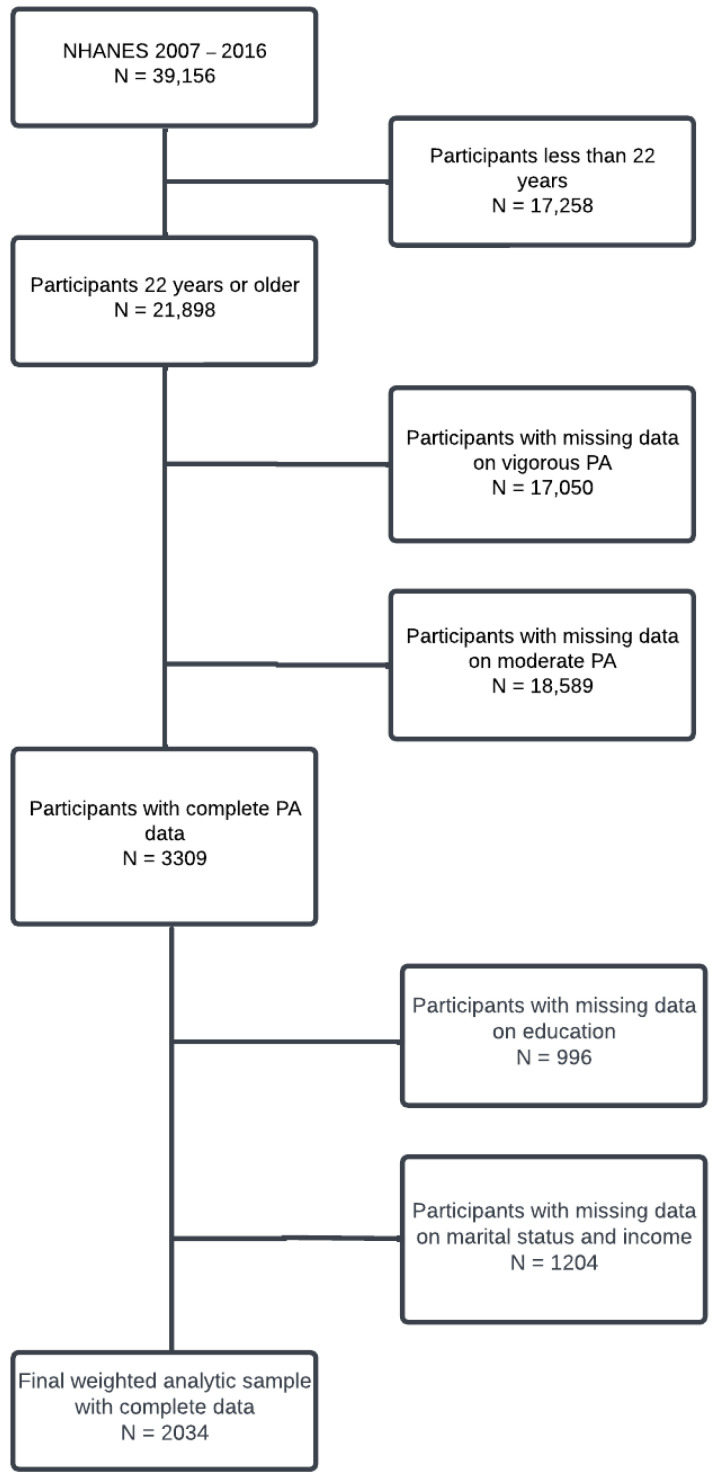
Algorithm used to define the study cohort.

Data were drawn from the 2007–2016 National Health and Nutrition Examination Survey (NHANES). NHANES is a stratified, national, multistage cross-sectional sample that evaluates the health and nutritional status of the U.S. civilian population [19,20]. NHANES uses complex probability sampling to select participants that represent a national sample, including oversampling for reliable estimates for population subgroups (i.e., non-Hispanic Black and low-income Whites) [19,20]. Data were collected in 2-year cycles beginning in 1999, which are still ongoing. NHANES participants completed at-home interviews via questionnaires along with a physical examination. Home interviews were used to collect self-reported demographic, socioeconomic, and health-related data, amongst other information. The present study was not reviewed by the Institutional Review Board as the data are de-identified and publicly accessible. Adults over the age of 22 who reported complete information for all the variables used in this study were selected for inclusion. Participants who were missing data on any of the study variables were excluded. The final study cohort was comprised of 2034 participants based on a complete case analysis (Figure 1).

### 2.2. Study Variables

#### 2.2.1. Moderate and Vigorous Sport, Fitness, and Recreational Physical Activity

Moderate and vigorous sport, fitness, and recreational physical activity were the primary outcome variables of this study. This physical activity was measured by two structured, open-ended questions via a questionnaire. Participants were initially asked whether they engaged in at least 10 min of moderate or vigorous sport, fitness, and recreational physical activities. They were informed that moderate physical activity was defined as any activity that causes small increases in breathing or heart rate and that vigorous physical activity was any activity that causes large increases in breathing or heart rate. Participants who responded “yes” to engaging in at least ten minutes of moderate and vigorous PA were then asked about the number of days in a typical week they engaged in that specific type of PA. These responses ranged from one to seven days a week. Finally, participants were specifically asked “How much time do you spend doing moderate-intensity sports, fitness or recreational activities on a typical day?” and “How much time do you spend doing vigorous-intensity sports, fitness or recreational activities on a typical day?” For data analysis, we created a composite variable to represent moderate and vigorous PA minutes per week. We took participants response to number of minutes in a typical day and multiplied it by participants’ response to the number of days they engaged in the activity in a typical week. The minutes of PA per week for each category was analyzed as a continuous variable.

#### 2.2.2. Number of Children in Household

Number of children in the household was the primary independent variable of interest for this study and was measured with two variables. The first was the number of children living in the participant’s household aged 5 years or younger and the second was the number of children living in the household who were between 6 and 17 years of age. Each variable was derived from a questionnaire item asking respondents to indicate the number of children for each respective age group. The response categories ranged from zero to three or more for children in the household five and younger, and zero to four or more for children in the household between 6 and 17 years of age. We modified both variables to have three response categories: no children, one child, and two or more children.

#### 2.2.3. Study Covariates

Covariates included seven demographic variables often associated with physical activity. Age was measured in years, and sex was a dichotomous variable that indicated whether a respondent classified themselves as male or female. Race was a categorical variable indicating whether respondents identified themselves as non-Hispanic Black, non-Hispanic white, Hispanic, or other. The marital status variable indicated whether respondents indicated that they were never married, living with a partner, married, separated, divorced, or widowed. Educational attainment was a categorical variable which had four responses: “completed less than 9 years of school”, “completed 9–11 years”, “completed 12 years”, and “completed more than 12 years of education”. Household income was a measure with which respondents indicated that their family’s total annual income fell within one of five categories, which included “less than $19,999”; “$20,000–$54,999”; “$55,000–$74,999”; “$75,000–$99,999”; or “$100,000 and above”. The final covariate was the total number of people reported in the participant’s household, with responses ranging from one to seven or more.

### 2.3. Statistical Analysis and Survey Design

Descriptive statistics were used to characterize study participants by age and number of children in the household. We report means and standard error for continuous variables and proportions for continuous variables. All means were weighted for the complex NHANES survey design. Statistical significance was assessed with two-tailed tests and α = 0.05. First, we ran separate ANOVAs to test for mean differences in self-reported physical activity and other covariates based on the number of children in the household aged 0–5 and 6–17.

Next, a multivariable regression analysis was conducted to evaluate if the number of children in the household aged 0–5 and 6–17 were significant predictors of self-reported sport, fitness, and recreational physical activity among US adults. Model 1 included adjustments for race, age, and sex; Model 2 added educational attainment and household income to Model 1; and Model 3 added marital status and total household size to Model 2. Four separate models were run for moderate and vigorous PA and by age group. Data from the regression models are reported at 95% confidence intervals (CIs), and associated *p*-value for the specific characteristic. We calculated the mean and SE for continuous variables. For categorical variables, we obtained the frequency and proportions. All statistical analyses were conducted using SAS software V.9.4 (SAS Institute, Cary, North Carolina, USA), SUDAAN software Release 11.0.3 (SUDAAN Statistical Software Center, Research Triangle Park, North Carolina, USA), *p*-values less than 0.05 were considered statistically significant.

## 3. Results

### 3.1. Baseline Characteristics

Baseline participant characteristics for adults with children aged 0–5 in the household are presented in Table 1. Adults with no children in the household aged 0–5 comprised 77% of the study cohort, 16% of the sample had one child in this age group, and 7% had two or more children aged 0–5 in the household. The mean (SE) age (years) for this group was 42 (0.5) and significantly differed by number of children. ANOVA analyses revealed significant differences between the number of children in the household aged 0–5 and self-reported minutes of vigorous physical activity per week (*p* < 0.001), race (*p* < 0.001), educational attainment (*p* < 0.001), marital status (*p* < 0.001), household size (*p* < 0.001), and income (*p* = 0.006). Overall, adults with more children aged 0–5 in the household tended to engage in fewer minutes of moderate and vigorous physical activity per week in the bivariate analyses.

Baseline participant characteristics for adults with children aged 6–17 in the household are presented in Table 2. Adults with no children in the household aged 6–17 comprised 64% of the study cohort, 17% of the sample had one child in this age group, 13% had two children 6–17, and 6% of the sample had three or more children. The mean (SE) age for this group was 42.3 (0.5) and significantly differed by number of children. ANOVAs analyses revealed significant differences between the number of children in the household aged 6–17 and race (*p* < 0.001), educational attainment (*p* < 0.001), marital status (*p* < 0.001), household size (*p* < 0.001), and income (*p* = 0.02). Overall, adults with more children aged 6–17 in the household tended to engage in fewer minutes of moderate and vigorous physical activity per week in the bivariate analyses.

### 3.2. Differences in Adult Moderate Physical Activity by Age and Number of Children

Results from the multivariable regression models for moderate sport, fitness, and recreational physical activity of adults with children in the household aged 0–5 are presented in Table 3. For moderate physical activity, there were no significant differences between the minutes of moderate PA per week between adults with no children or just one child compared to those with two or more children in this age group. While insignificant, adults with one child engaged in about twenty-two fewer minutes of moderate PA per week than those with no children, and adults with two children engaged in about twenty-six fewer minutes of moderate PA per week than those with no children after adjusting for all covariates (model 3).

Results from the multivariable regression models for moderate sport, fitness, and recreational physical activity of adults with children in the household aged 6–17 are presented in Table 4. For moderate physical activity, there were no significant differences between the amount of activity between adults with no children, just one child, two children, or three or more children in this age group after adjusting for the covariates (model 3). While insignificant, adults with one child engaged in about twenty-seven fewer minutes of moderate PA per week than those with no children, adults with two children engaged in about thirty fewer minutes of moderate PA per week than those with no children, and adults with three or more children in this age group engaged in nearly forty fewer minutes of moderate PA per week after adjusting for all covariates (model 3).

### 3.3. Differences in Adult Vigorous Physical Activity by Age and Number of Children

Results from the multivariable regression models for vigorous sport, fitness, and recreational physical activity of adults with children in the household aged 0–5 are presented in Table 5. For vigorous physical activity, there was a significant difference between the minutes of vigorous PA per week between adults with no children or just one child compared to those with two or more children in this age group (*p* < 0.05) after adjusting for all covariate (model 3). Adults with two or more children engaged in more than eighty fewer minutes of weekly vigorous PA activity compared to those with no children. Moreover, adults with one child in this age group engaged in about eight fewer minutes of vigorous PA compared to those with no children (model 3).

Results from the multivariable regression models for vigorous sport, fitness, and recreational physical activity of adults with children in the household aged 6–17 are presented in Table 6. There was a significant difference between the minutes of vigorous PA per week between adults with no children compared to those with three or more children in this age group (*p* < 0.05) even after adjusting for age, sex, race, educational attainment, and income (model 2). In this model, adults with three or more children engaged in fifty fewer minutes of weekly vigorous PA compared to those with no children. There were no significant differences in weekly vigorous PA between those with no children, one child, and two children in this age group across all models.

## 4. Discussion

This study examined the association between adult moderate and vigorous sport, fitness, and recreational physical activity and the number of and age of children in their household. Our hypotheses were partially supported in that we found differences in adult weekly minutes of vigorous sport, fitness, and recreational PA based on the number of and age of children in the household after controlling for several covariates.

The first major finding of this study was adults with two or more children aged 0–5 in the home engaged in significantly less vigorous physical activity compared to those with no children or just one child. Similarly, adults with three or more children in the household aged 6–17 engaged in significantly less weekly vigorous PA than those with none, one, or just two children in this age group. Additionally, there were no significant differences in adult weekly minutes of moderate PA regardless of the number of children aged 0–5 or 6–17.

These findings are aligned with previous studies that found differences in different types of physical activity between parents and non-parents of younger and older children [4,6,14]. For example, Berge et al. [4] found that parents of infant-age (less than one) children engaged in roughly one less hour of MVPA per week compared to individuals without children. Our results highlight a similar trend in that adults with two or more children under five tended to engage in eighty fewer minutes of vigorous PA in a typical week. Similar results have been found in Canadian samples across both younger and adolescent-age children [6,14]. Moreover, in the present study, we only found differences in weekly vigorous PA and not moderate PA.

A key difference In our results was a similar association between the impact of children on PA for both mothers and fathers. Specifically, sex was not a significant predictor of MVPA in our regression models, although several studies have found this relationship to be stronger for mothers compared to fathers [6,14,16]. We posit that the difference in our findings could be due to the difference in the measurement of PA. Those studies objectively measured and compared total daily PA, while we exclusively considered self-reported sport, fitness, and recreational physical activity. Thus, while fathers may be less impacted than mothers in their daily total PA as those studies found, perhaps the primary differences between mothers and fathers could lie in moderate PA, as we found no differences in vigorous PA between mothers and fathers in the current study. Future studies should further explore variances in types of PA by mothers and fathers based on the number and age of children in the home.

While the interest and knowledge of correlates and determinants of physical activity has drastically increased in the past few decades, we posit that the translation of this knowledge into effective interventions and policies have moved at a much slower pace, especially in supporting the physical activity of adults with multiple young children. Ding et al. [21] support this assertion in their recent article and indicate that “it is uncertain to what degree the growing literature has led to better-informed interventions and policies” [21].

From an intervention standpoint, a vast majority of the family-based physical activity interventions have included only familial dyads (i.e., parent(s) and one child). An extensive review of the 49 studies included in a systematic review of family-based PA interventions by Brown et al. [22] highlighted the lack of inclusion of parents with multiple children in this body of literature. For example, the Girl’s health Enrichment Multi-site Studies (GEMS) is a child- and parent-targeted intervention that has been effective for families across several regions in the United States [23,24,25,26]. Moreover, in each iteration of the GEMS studies, they only included data for the parents and one child, even if they had multiple children in the household. Similarly, A Family Affair, Adventuras Para Ninos, Healthy Choices Intervention, Active Balance Childhood study, BOUNCE, and GET FIT are all other successful family-based PA interventions reporting the data of exclusively family dyads or triads across a variety of ages and ethnicities, and do not exclusively report the data of parents/guardians with multiple children [27,28,29,30,31,32]. A few studies, such as Children, Parents, and Pets (CPET), America on the Move (AOM), and Healthy Dads, Healthy Kids, did include some families that had multiple children in their studies, but an overwhelming majority of families were made up of dyads [33,34,35]. This review left us with a lingering question: what has been done to support parents with more than one young child? Parental role modeling of PA has consistently been associated with young children’s PA [8,9,11], which implies that targeting these parents and including multiple children in these interventions may be an effective tool to impact several children at once. There is a need for future family-based PA intervention studies with a wider inclusion, and reporting, of children and adolescents rather than solely dyadic studies.

From a population health perspective, there appears to be a lack of programs and policies that have been put in place specifically for the support of physical activity of parents with multiple young children. The Lancet physical activity series posited that policy changes may have the most profound impacts on positive behavioral changes for population health [36]. Others also support this assertion and highlight a need to implement policy strategies that are more likely to lead to sustainable population-level behavior change [21,37]. Accordingly, we believe health-related policy strategies in the workplace may be an effective agent of change in supporting the physical activity of this group, as this is likely where they spend a majority of their time outside of parenting [38,39]. Such worksite wellness programs already exist, but the overall effectiveness of these programs is inconclusive, which may largely be attributed to the implementation strategies of these programs and policies [40,41]. For example, Smith-McLallen et al. [42] examined the effectiveness of a worksite walking intervention for six small Philadelphia-based companies who were independent wellness partners of Blue Cross Blue Shield. The employees in this study increased their average daily steps by over 700 steps and lost an average of three pounds over a 9-month period. Moreover, they highlighted the inclusion of incentives, feedback, competitive challenges, and monthly workshops as implementation strategies to increase employee engagement in these programs. Bensa and Sirok [43] most recently emphasized the need to identify determinants of participation in these programs to better target specific subgroups who may not participate in these programs. We believe such a focus could be beneficial in targeting the participation of parents with several young children. One strategy that may appease to this group is an incentive that supports engagement in short bouts of PA throughout the workday. Not only could this strategy increase their workday PA engagement, but it could potentially translate to short at-home exercise when parents may only have short periods of free time and often highlight time as a common barrier to exercise. Research suggests short bouts of MVPA accumulated across the entire day is associated with health outcomes, including all-cause mortality [44].

### Strengths and Limitations

A strength of this study includes the use of a nationally representative sample of US adults, as well as the inclusion of several sociodemographic factors in our analysis. How-ever, this study is not without limitations. First, we used cross-sectional data from the NHANES interviews and assessments. Thus, the PA data of the adults in this study were self-reported measures. Accordingly, we were not able to separate sport, fitness, and recre-ational physical activities as their own categories of PA. In addition, a majority of the sam-ple were White adults with no children in the household, which may impact the generali-zability of these findings. Finally, we understand that there are many other factors (e.g., social, psychological, and environmental) associated with an individual’s engagement in PA that were not accounted for in this study.

## 5. Conclusions

Overall, the presence of multiple young children and adolescents in the home significantly reduced the likelihood of parents engaging in vigorous sport, fitness, and recreational physical activities. These findings highlight an apparent need to support the PA of parents with multiple children. To our knowledge, there is currently very little published research that has focused on increasing vigorous PA among adults with multiple children in the household. Therefore, our study will make a significant contribution to the growing body of literature of factors that influence parental PA behaviors. According to these findings, there appears to be a need to explore this association with data that include more parents with children across a wide age-range to better understand this relationship.

## Figures and Tables

**Table 1:** Demographic, socioeconomic, and physical activity characteristics of participants by number of children aged 0–5 in the household.

Characteristics	All	No Children	1 Child	2 or More Children	*p*-Value
N (%)	2034	1562 (77%)	334 (16%)	138 (7%)	
Moderate PA (mins/week) (mean) (SE)	197.9 (5.7)	202.4 (6.1)	181.4 (14.2)	175.3 (17.0)	0.23
Vigorous PA (mins/week) (mean) (SE)	219.0 (7.5)	239.4 (8.5)	235.9 (20.0)	160.3 (8.5)	<0.0001
Age (mean) (SE)	42.3 (0.5)	44.3 (0.6)	34.9 (0.5)	32.7 (0.6)	<0.0001
Sex					0.46
Female (%)	895 (45.5%)	670 (36.0%)	163 (6.8%)	62 (2.7%)	
Male (%)	1139 (55.0%)	892 (44.0%)	171 (7.2%)	76 (3.4%)	
Race (%)					<0.0001
Non-Hispanic Black	441 (10.0%)	335 (7.5%)	79 (1.8%)	27 (0.6%)	
Non-Hispanic White	830 (70.4%)	663 (58.6%)	115 (8.1%)	52 (3.7%)	
Hispanic	399 (8.3%)	283 (7.6%)	81 (2.7%)	35 (1.1%)	
Other	364 (8.3%)	281 (6.3%)	59 (1.4%)	24 (0.6%)	
Education (%)					<0.0001
<9 years	71 (1.9%)	49 (1.3%)	15 (0.4%)	7 (0.2%)	
9–11 years	129 (4.1%)	80 (2.4%)	37 (1.3%)	12 (0.3%)	
12 years	309 (13.2%)	232 (10.3%)	60 (2.3%)	17 (0.6%)	
Some college or Associate degree	610 (28.5%)	458 (22.4%)	106 (4.4%)	46 (1.7%)	
College graduate or above	915 (52.2%)	743 (43.4%)	116 (5.6%)	56 (3.2%)	
Marital status (%)					<0.0001
Married	1196 (64.0%)	861 (48.4%)	234 (10.7%)	101 (5.0%)	
Widowed	62 (2.0%)	57 (1.9%)	3 (0.1%)	2 (0.04%)	
Divorced	195 (8.9%)	166 (8.0%)	23 (0.7%)	6 (0.2%)	
Separated	47 (1.7%)	36 (1.4%)	8 (0.3%)	3 (0.07%)	
Never married	360 (15.0%)	321 (13.8%)	31 (1.0%)	8 (0.3%)	
Living with partner	174 (8.4%)	121 (6.6%)	35 (1.3%)	18 (0.5%)	
Annual income (%)					0.006
<$19,999	223 (6.9%)	171 (5.2%)	32 (1.0%)	20 (0.7%)	
$20,000–$54,999	638 (25.4%)	456 (18.6%)	124 (4.7%)	58 (2.2%)	
$55,000–$74,999	249 (12.4%)	203 (10.4%)	36 (1.6%)	10 (0.4%)	
$75,000–$99,999	248 (11.9%)	186 (9.4%)	46 (1.8%)	16 (0.8%)	
>$100,000	676 (43.4%)	546 (36.4%)	96 (5.0%)	34 (1.9%)	
Total of people in household (SE)	3.0 (0.05)	2.7 (0.04)	4.1 (0.08)	4.7 (0.08)	<0.0001

**Table 2:** Demographic, socioeconomic, and physical activity characteristics of participants by number of children aged 6–17 in the household.

Characteristics	All	No Children	1 Child	2 Children	3 or More Children	*p*-Value
N (%)	2034	1297 (64%)	343 (17%)	256 (13%)	138 (6%)	
Moderate PA (mins/week) (mean) (SE)	197.9 (5.7)	204.4 (6.7)	186.1 (12.7)	182.5 (17.5)	185.6 (17.6)	0.42
Vigorous PA (mins/week) (mean) (SE)	219.0 (7.5)	237.2 (8.3)	234.1 (18.0)	234.5 (21.0)	192.9 (14.9)	0.08
Age (SE)	42.3 (0.5)	43.1 (0.7)	40.7 (0.7)	40.5 (0.6)	39.5 (0.7)	0.004
Sex (%)						0.27
Female	895 (45.5%)	537 (29.8%)	169 (7.2%)	123 (5.9%)	66 (2.6%)	
Male	1139 (54.5%)	760 (37.1%)	174 (7.6%)	133 (7.3%)	72 (2.5%)	
Race (%)						<0.0001
Non-Hispanic Black	441 (10.0%)	283 (6.3%)	81 (1.9%)	39 (0.9%)	38 (0.8%)	
Non-Hispanic White	830 (70.4%)	579 (49.4%)	103 (8.8%)	100 (9.3%)	48 (3.0%)	
Hispanic	399 (11.4%)	200 (5.6%)	89 (2.6%)	70 (2.1%)	40 (1.1%)	
Other	364 (8.3%)	235 (5.6%)	70 (1.5%)	47 (0.9%)	12 (0.2%)	
Education (%)						<0.0001
<9 years	71 (1.9%)	32 (0.8%)	17 (0.5%)	14 (0.4%)	8 (0.2%)	
9–11 years	129 (4.1%)	57 (2.0%)	32 (0.8%)	23 (0.8%)	17 (0.5%)	
12 years	309 (13.2%)	185 (8.4%)	60 (2.6%)	30 (1.0%)	34 (1.2%)	
Some college or Associate degree	610 (28.5%)	389 (18.6%)	108 (4.3%)	78 (4.3%)	35 (1.3%)	
College graduate or above	915 (52.2%)	634 (37.2%)	126 (6.6%)	111 (6.5%)	44 (1.9%)	
Marital status (%)						<0.0001
Married	1196 (64.0%)	670 (38.4%)	224 (10.6%)	195 (10.7%)	107 (4.3%)	
Widowed	62 (2.0%)	46 (1.5%)	11 (0.3%)	4 (0.2%)	1 (0.01%)	
Divorced	195 (8.9%)	140 (6.6%)	28 (1.2%)	18 (0.8%)	9 (0.3%)	
Separated	47 (1.7%)	26 (1.0%)	11 (0.4%)	7 (0.3%)	3 (0.07%)	
Never married	360 (15.0%)	310 (13.8%)	35 (0.9%)	10 (0.3%)	5 (0.1%)	
Living with partner	174 (8.4%)	105 (5.6%)	34 (1.4%)	22 (1.0%)	13 (0.4%)	
Annual income (%)						0.09
<$19,999	223 (6.9%)	160 (5.0%)	36 (1.1%)	15 (0.6%)	12 (0.2%)	
$20,000–$54,999	638 (25.4%)	383 (17.3%)	122 (3.7%)	76 (2.6%)	57 (1.7%)	
$55,000–$74,999	249 (12.4%)	171 (9.4%)	35 (1.3%)	29 (1.3%)	14 (0.5%)	
$75,000–$99,999	248 (11.9%)	164 (7.8%)	40 (1.9%)	28 (1.4%)	16 (0.8%)	
>$100,000	676 (43.4%)	419 (27.4%)	110 (6.9%)	108 (7.2%)	39 (1.9%)	
Total of people in household (SE)	3.0 (0.05)	2.3 (0.04)	3.8 (0.07)	4.4 (0.06)	5.7 (0.08)	<0.0001

**Table 3:** Number of children aged 0–5 in household regressed on weekly minutes of moderate physical activity.

	Unadjusted	Adjusted		
Parameter	Estimate (SE)	Model 1	Model 2	Model 3
No children	202.4 (6.1)	206.0 (6.4)	236.9 (11.0)	236.9 (10.9)
1 child	−21.0 (14.4)	−17.1 (14.3)	−20.6 (13.6)	−21.6 (13.7)
2 or more children	−27.1 (18.9)	−22.6 (19.7)	−24.6 (18.6)	−26.1 (19.2)

Model 1 adjusts for age, sex, and race. Model 2 adds education and income to model 1. Model 3 adds marital status and total number of people in the household to model 2.

**Table 4:** Number of children aged 6–17 in household regressed on weekly minutes of moderate physical activity.

	Unadjusted	Adjusted		
Parameter	Estimate (SE)	Model 1	Model 2	Model 3
No children	204.4 (6.7)	208.9 (7.1)	240.3 (11.2)	242.8 (10.6)
1 child	−18.4 (13.9)	−18.4 (3.7)	−20.4 (13.4)	−27.0 (5.6)
2 children	−22.0 (18.8)	−21.6 (18.5)	−20.8 (19.0)	−30.5 (19.4)
3 or more children	−18.9 (18.7)	−19.1 (8.5)	−23.0 (17.6)	−39.1 (23.8)

Model 1 adjusts for age, sex, and race. Model 2 adds education and income to model 1. Model 3 adds marital status and total number of people in the household to model 2.

**Table 5:** Number of children aged 0–5 in household regressed on weekly minutes of vigorous physical activity.

	Unadjusted	Adjusted		
Parameter	Estimate (SE)	Model 1	Model 2	Model 3
No children	239.4 (8.5)	247.9 (7.0)	268.1 (13.0)	267.7 (12.8)
1 child	−3.5 (21.7)	−15.0 (21.9)	−15.9 (19.7)	−7.9 (21.6)
2 or more children	−79.1 (15.8) *	−93.0 (16.7) *	−96.6 (16.3) *	−84.1 (19.0) *

* denotes a *p*-value < 0.05. Model 1 adjusts for age, sex, and race. Model 2 adds education and income to model 1. Model 3 adds marital status and total number of people in the household to model 2.

**Table 6:** Number of children aged 6–17 in household regressed on weekly minutes of vigorous physical activity.

	Unadjusted	Adjusted		
Parameter	Estimate (SE)	Model 1	Model 2	Model 3
No children	237.2 (8.3)	244.2 (7.0)	264.4 (16.3)	257.4 (16.4)
1 child	−3.1 (17.3)	−6.4 (18.0)	−5.6 (19.0)	12.8 (21.8)
2 children	−2.7 (22.5)	−4.7 (22.7)	−1.0 (23.2)	26.0 (25.1)
3 or more children	−44.3 (16.7) *	−51.0 (16.6) *	−50.4 (17.6) *	−5.4 (23.3)

* denotes a *p*-value < 0.05. Model 1 adjusts for age, sex, and race. Model 2 adds education and income to model 1. Model 3 adds marital status and total number of people in the household to model 2.

## Data Availability

All the data used in the study are publicly available from the Centers for Disease Control and Prevention.
